# The gap between trials and reality

**DOI:** 10.1093/bjsopen/zraf021

**Published:** 2025-03-19

**Authors:** Matthew J Lee

**Affiliations:** Department of Applied Health Sciences, College of Medicine & Health, University of Birmingham, Birmingham, UK

Of 6300 people who apply to be NASA astronauts, eight will be selected for training, giving a 0.1% success rate^1^. One cannot use the achievements of astronauts to make assumptions about the achievements of the general population. Similarly, given that randomized clinical trials (RCTs) can reflect highly selected populations, it may be difficult to know whether the group studied is comparable to the general, unselected population we serve. The review by Cutteridge *et al.*^2^ brings this issue into sharp focus for the vascular surgery community and, by extension, the wider academic surgery community. This review demonstrates that the patients who are entered into trials have different characteristics compared to those with treatment documented in a national registry. It also shows highly variable reporting between trials, and between trials and the national registry.

Do these discrepancies matter? If one wishes to know whether an intervention is effective in a very narrow sense, then perhaps not. However, for the surgeon who lives in a diverse world with an ageing population, or for the health economist who attempts to understand the cost-effectiveness of an intervention for their population, then this is a very real problem. One might harbour suspicions that trials with a younger age profile of patients might see favourable outcomes *versus* the older population that exist in the real world. This could lead to implementation of interventions that do not benefit our patients and may even lead to harm. The review also highlights that ethnicity is poorly reported, and consequently such diversity may be underrepresented in trial results. With evidence that ethnicity is associated with the prevalence and presentation of peripheral arterial disease^3^, this is surely a factor that requires further attention through robust reporting.

Therefore, we should take two key actions. First, we must make efforts to deliver pragmatic trials with broad recruitment criteria. Vascular surgery is an exemplar of a specialty linked to social inequality^4^, and no effort should be spared in engaging this underserved population. We must consider efforts to maximize inclusivity as an opportunity to maximize yield of knowledge, rather than an obstruction. This should include strategies to recruit older patients, those with limited ability to speak or read the primary national language, and people from the various ethnic groups we serve. In the UK, the major health research funder has now mandated inclusivity strategies as a condition of funding. This attitude should not be constrained by geography.

Second, selective reporting of characteristics is not limited to vascular surgery. This has led to the proposal of standardizing patient and disease descriptors for conditions using a ‘core descriptors’ approach, with examples generated across a range of conditions. There is a need to develop this idea for vascular surgery.

In 1994, Doug Altman highlighted the scandal of money wasted on poor-quality research, linked in part to unrepresentative samples^5^. Thirty years later, Cutteridge and colleagues have highlighted that this remains true for vascular surgery. Will a 2055 editorial still find this key aspect of research wanting?

## References

[1] NASA . NASA Receives Second Highest Number Of Astronaut Applications. *NASA*. https://www.nasa.gov/news-release/nasa-receives-second-highest-number-of-astronaut-applications/ (accessed 3 February 2025)

[2] Cutteridge J, Barsby J, Hume S, Lemmey HAL, Lee R, Bera KD. External validity of randomized clinical trials in vascular surgery: systematic review of demographic factors of patients recruited to randomized clinical trials with comparison to National Vascular Registry. BJS Open 2025; doi: 10.1093/bjsopen/zrae156 PMC11921775 40105904

[3] Vitalis A, Lip GYH, Kay M, Vohra RK, Shantsila A. Ethnic differences in the prevalence of peripheral arterial disease: a systematic review and meta-analysis. Expert Rev Cardiovasc Ther 2017;15:327–338 28290228 10.1080/14779072.2017.1305890

[4] Pande RL, Creager MA. Socioeconomic inequality and peripheral artery disease prevalence in US adults. Circ Cardiovasc Qual Outcomes 2014;7:532–539 24987053 10.1161/CIRCOUTCOMES.113.000618 PMC4219271

[5] Altman DG . The scandal of poor medical research. BMJ 1994;308:283–284 8124111 10.1136/bmj.308.6924.283 PMC2539276

